# Checklist of the coral fish fauna of Xisha Islands, China

**DOI:** 10.3897/BDJ.9.e63945

**Published:** 2021-03-08

**Authors:** Shuting Qiu, Bin Chen, Jianguo Du, Kar-Hoe Loh, Jianji Liao, Xinming Liu, Wen Yang

**Affiliations:** 1 Third Institute of Oceanography, Ministry of Natural Resources, Xiamen, China Third Institute of Oceanography, Ministry of Natural Resources Xiamen China; 2 Fujian Provincial Key Laboratory ofMarine Ecological Conservation and Restoration, Xiamen, China Fujian Provincial Key Laboratory ofMarine Ecological Conservation and Restoration Xiamen China; 3 Institute of Ocean and Earth Sciences, University of Malaya, Kuala Lumpur, Malaysia Institute of Ocean and Earth Sciences, University of Malaya Kuala Lumpur Malaysia; 4 Guangxi University of Chinese Medicine, Guangxi, China Guangxi University of Chinese Medicine Guangxi China

**Keywords:** Ichthyofauna, Coral Fish Diversity Index (CFDI), Xisha Islands, newly-recorded species

## Abstract

**Background:**

The Xisha Islands are composed of the Yongle Islands and the Xuande Islands in Hainan Province, China. It has one of the highest species diversity in the world and is also a typical oceanic distribution area of coral reefs globally. The ichthyofauna of the Xisha Islands were recorded by underwater visual census in May 2019 and July 2020. The survey data were combined with previous records of species into the checklist of the Xisha Islands presented herein. A total of 691 species, belonging to 24 orders and 97 families, was recorded. The major families were Labridae, Pomacentridae, Serranidae, Chaetodontidae, Hexanchidae, Lutjanidae, Scaridae, Gobiidae, Scorpaenidae and Carangidae. In this study, the Coral Fish iversity Index (CFDI) of six families (Chaetodontidae, Pomacanthidae, Pomacentridae, Labridae, Scaridae and Acanthuridae) was 229, indicating 756 coral fishes. In terms of the IUCN Red List, one species is Critically Endangered (*Glyphis gangeticus*), six species are Endangered (*Stegostoma fasciatum*, *Aetomylaeus maculatus*, *Aetomylaeus vespertilio*, *Epinephelus akaara*, *Cheilinusundulatus* sp. and *Xiphias gladius*), 16 species are Vulnerable, and 13 species are Near Threatened in the Xisha Archipelago, so conservation should be strengthened in this area in the future.

**New information:**

One species is a new record for China (*Dischistodus pseudochrysopoecilus*) and 23 species are newly found in the Xisha Islands.

## Introduction

The Xisha Archipelago is in the midwest of the South China Sea and it is at the northern margin of the Coral Triangle, which has the highest biodiversity in the world (Allen 2008). The Xisha Islands comprise the Yongle Islands and the Xuande Islands and begins in the north from the Beijiao Atoll and ends at the Songtao Bank in the south; Xidu Bank is in the east and Zhongjian Island in the west. Altogether, there are 29 islands, sandbars and four atolls.

The coral reefs of the Xisha Islands are the oldest and most primitive in China. It is also the birthplace of the coral reef ecosystem in the coastal areas of China (Huang et al. 2020). The Xisha Coral Reef Ecological Monitoring Zone was established in 2005 by the State Oceanic Administration of China to monitor and assess the health of coral reefs. The coral reef coverage declined from 53.8% in 2007 to 5.44% in 2016, due to anthropogenic activities, outbreak of crown-of-thorns starfish, coral diseases, typhoons and global warming (Li et al. 2017).

Several studies have focused on the fishes in the Xisha Islands. For example, 261 species belonging to 48 families of coral reef fishes were found in Beijiao Atoll, Yongxing Island, Huaguang Atoll and five other islands by gillnet and angling (Sun et al. 2004). A total of 146 species belonging to 31 families of fish was reported from the main reefs in Xisha Islands by bottom gillnet (Wang et al. 2011). There were 119 species belonging to 73 genera and 30 families of coral reef fish species reported in the Xisha Islands by underwater visual censuses and 643 species were recorded in combination with other previous studies (Gao et al. 2014). However, several new studies have been published. For example, in Zhaoshu Island, a total of 58 coral reef fish species was recorded, belonging to one class, three orders, 18 families and 37 genera by underwater visual censuses (Yang et al. 2018).The diversity of reef fishes declined from 3.10/m^2^ in 2005 to 1.23/m^2^ in 2013, due to reef degradation and overfishing (Li et al. 2017). The fish list of the Xisha Islands remains incomplete and taxonomic revisions are urgently needed to provide scientific support for follow-up research and protection of reef fishes in the Xisha Islands, especially against the background of intense human activity and rapid global change.

## Materials and methods

**Figure 1.**
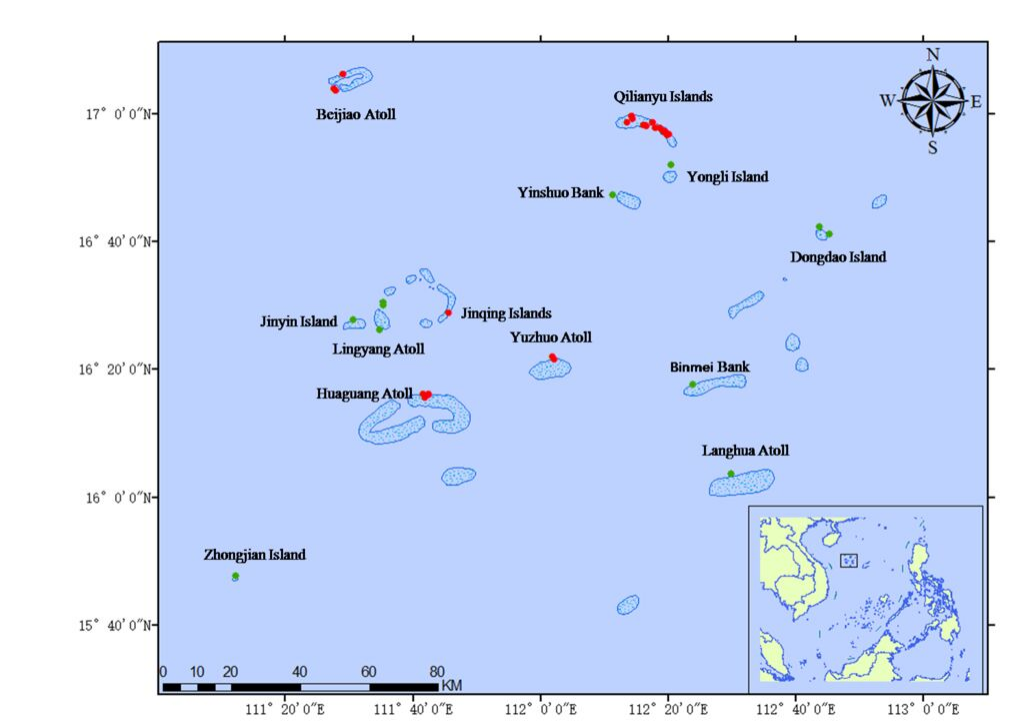
The map showing the survey locations.

In May 2019 and July 2020, a total of 27 sites in the Xisha Islands (Fig. 1) was investigated by underwater visual censuses and more than 50 dives were performed, using a Canon 5D4, together with Seacam 150D and Sea&Sea YS-D2 flashlights. The photos were taken ranging from 5 to 30 m. The species were identified according to the following resources: Reef fishes of the East Indies (Allen and Erdmann 2012), Coral reef atlas of Xisha Islands (Huang 2018), Reef fish identification of Nansha Islands (Fang and Lv 2019), Coral reef fishes of the South China Sea (Fu 2014) and FishBase (Froese and Pauly 2020, Fig. 1)

The Coral Fish Diversity Index (CFDI) is an evaluative index proposed by Allen (1988) to measure the diversity of coral reef fishes using the following formula: Total fish fauna = 3.39 (CFDI) - 20.595 used by for areas under 2,000 km. The CFDI is based on the total number of species in each of the six indicator families (Acanthuridae, Chaetodontidae, Labridae, Pomacanthidae, Pomacentridae and Scaridae). All selected families are crucial parts of reef communities, widely distributed and firmly related to coral reef ecosystems.

This checklist has been arranged in the evolutionary order of class, order, family and species and families are arranged in the evolutionary order of genera and then species name.The newly-recorded species in the Xisha Archipelago are marked with r11 only in the Table. For habitats types, these abbreviations have been used: reef associated (RFA); brackish (BRA); demersal (DEM); amphidromous (AMP); pelagic (PEL); bathydemersal (BAD); benthopelagic (BEP); bathypelagic (BAP); pelagic-neritic (PE); oceanodromous (OD).

The IUCN status is indicated as: Critically Endangered (CE); Endangered (EN); Vulnerable (VU); Near Threatened (NT); Lower Risk (LR); Least Concern (LC). Threat to humans: Other; Traumatogenic; Poisonous to eat; Venomous; Reports of ciguatera poisoning.

There were 197 species belonging to 28 families found in this survey, of which, one species represented a new record in China (*Dischistodus pseudochrysopoecilus*) and 23 species represent new records in the Xisha Islands (Table 1), combined with eight previous studies by Gao et al. (2014) and Sun et al. (2004)Li et al. 2017, Li et al. 2007, Wang 1981, Wang et al. 2011, Yang et al. 2018, Zeng 2004. In total, 690 species were recorded from the Xisha Islands, belonging to 24 orders and 97 families. The order Perciformes was the most dominant order, with 496 species belonging to it. The most dominant family was the family Labridae, which had 75 species; followed by Pomacentridae with 63 species; the third, fourth and and fifth largest families were Serranidae, Chaetodontidae and Hexanchidae, which had 42 species, 39 species and 29 species, respectively. Other major families were Lutjanidae and Scaridae, which both had 26 species, Gobiidae had 22 species; Scorpaenidae and Carangidae had 16 species (Table 1).

Other abbreviations and references include: Institute, South China Sea Fisheries 1979 [r1], Li et al. 2007 [r2], Sun et al. 2004 [r3], Zeng 2004 [r4], Wang 1981 [r5], Gao et al. 2014 [r6], Wang et al. 2011 [r7], Yang et al. 2018 [r8], Huang 2018 [r9], Fu 2014 [r10] and the current study [r11].

## Taxon treatments

### Dischistodus pseudochrysopoecilus

Allen & Robertson, 1974

B2B8DF9F-1E8A-5AA9-B948-9672E6B5BDF3

Dischistodus pseudochrysopoecilus Common name: Monarch damsel

#### Materials

- **Type status:** Holotype. **Location:** island: Xisha Island; country: China; stateProvince: Hainan

#### Taxon discussion

**Figure 2.**
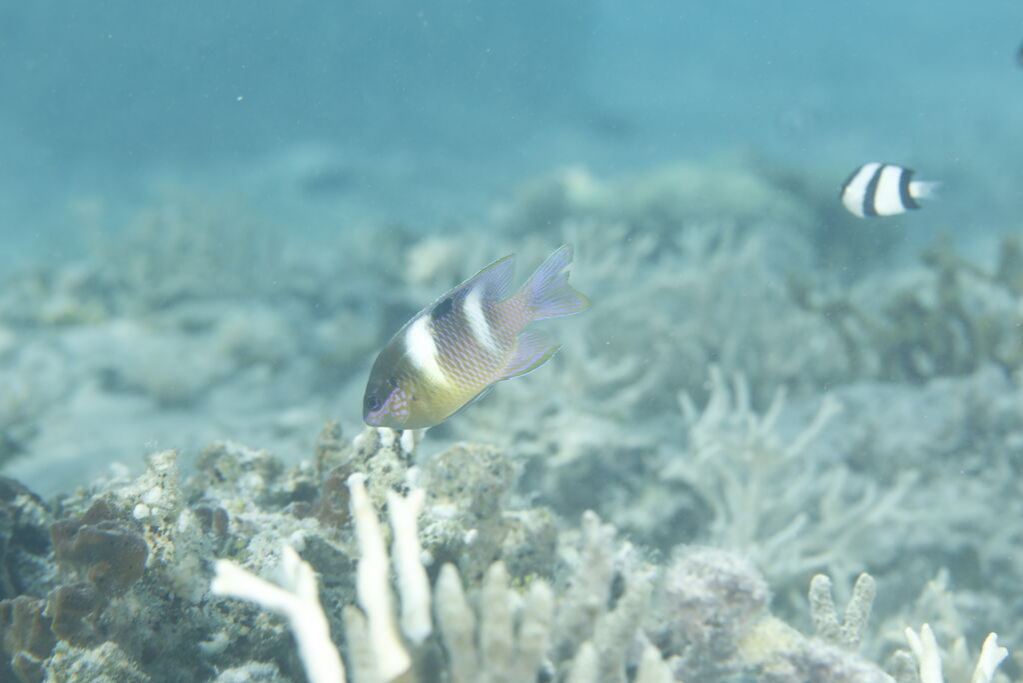
*Dischistodus pseudochrysopoecilus* — a newly-recorded species in China

Maximum size of 18 cm Fig. 2. Dark brown (nearly black) with blue streak on each scale, blue lines and spots on head and white spot on middle of upper back. Coral thickets interspersed with open sand or dead coral in 1-5 m (Allen and Erdmann 2012).

What we captured underwater in Xisha Islands is the state of the juvenile fish, which is very different from the adult fish. We have checked the main domestic fish checklists (Liu 2008, Chen and Zhang 2015, Shen and Wu 2011) and relevant literature, but there is no record of this species. Therefore, we believe it is a newly-recorded species in China.

## Discussion

According to previous research, the order Perciforms is the most dominant order in the Xisha Islands (Li et al. 2007) and the major families in the Xisha Islands are Labridae and Pomacentridae (Gao et al. 2014) which concur with our study. Combined with studies over the years, in the past 20 years, *Hyperoglyphe perciformis* has been the dominant species in both underwater visual censuses and gillnet surveys.

The CFDI value for the Xisha Archipelago was 229, such that we could estimate put it into the formula proposed by Allen 1988, we estimated that Xisha Islands had 756 coral reef fishes. However, only 690 species were found, which is relatively comprehensive, but there is still a need for more surveys in different seasons within different sites in the future.

In term of the IUCN Red List Anonymous (2020a), one species is Critically Endangered (*Glyphis gangeticus*); six species are Endangered (*Stegostoma fasciatum*, *Aetomylaeus maculatus*, *Aetomylaeus vespertilio*, *Epinephelus akaara*, *Cheilinus undulatus* and *Xiphias gladius*), 16 species are Vulnerable (*Nebrius ferrugineus*, *Alopias vulpinus*, *Carcharhinus albimarginatus*, *Carcharhinus falciformis*, *Carcharhinus longimanus*, *Hemigaleus microstoma*, *Sphyrna lewini*, *Rhina ancylostoma*, *Rhynchobatus djiddensis*, *Taeniurops meyeni*, *Urogymnus asperrimus*, *Epinephelus fuscoguttatus*, *Plectropomus areolatus*, *Thunnus obesus*, *Oxymonacanthus longirostris* and *Sphoeroides pachygaster*) and 13 species are Near Threatened in the Xisha Archipelago (*Aetobatus narinari, Isurus oxyrinchus*, *Atelomycterus marmoratus*, *Carcharhinus amblyrhynchoides*, *Carcharhinus limbatus*, *Galeocerdo cuvier*, *Prionace glauca*, *Scoliodon laticaudus*, *Triaenodon obesus*, *Hexanchus griseus*, *Chaetodon trifascialis*, *Choerodon schoenleinii* and *Thunnus albacares*). Thus, policy-makers and scientists should pay more attention to these species, by controlling coral reef degradation and overfishing and conducting coral reef restoration to strengthen the conservation of the fishes and the whole reef ecosystem. (Du et al. 2019). According to the data, 5.25% of the fish species are in the IUCN Red List, which is close to the number of the Redang Islands in Malaysia, at 5.1%.

Reef associated fish (FRA) are the dominant type in the Xisha Islands, which have 500 species. They constitute 83% of the total fish. Other types of fishes make up less than 5% of the total fish in the Xisha Islands.

According to Pauly et al. (2002), a trophic level of more than 3.5 is considered a high-grade carnivorous fish and nearly half (306 species) of all fishes in the Xisha Islands belong to this type.

Intriguingly, we found some species (for example: *Scolopsis aurata*) that were recorded in the Xisha Islands (Zeng 2004), but according to the Computer-Generated Native Distribution Map from Fishbase, this species currently is distributed in the Indian Ocean: Maldives, Sri Lanka and southern Indonesia. This may support the hypothesis of species shifting northwards, but more research is needed.

## Supplementary Material

XML Treatment for Dischistodus pseudochrysopoecilus

## Figures and Tables

**Table 1.** Checklist of the coral fish fauna of the Xisha Islands, China.

Subclass, Order, Family, Genus, and species	Habitat types	Threat to humans	IUCN status	Trophic level	Other remarks
Class Chondrichthyes					
Order Chimaeriformes					
Family Chimaeridae					
Genus *Chimaera*					
*Chimaera phantasma* Jordan & Snyder, 1900	BAD			3.5	r1
Order Orectolobiformes					
Family Orectolobidae					
Elasmobranchii					
Order Orectolobiformes					
Genus *Stegostoma*					
*Stegostoma fasciatum* (Hermann, 1783)	RFA	Traumatogenic	EN	3.1	r1
Family Ginglymostomatidae					
Genus *Nebrius*					
*Nebrius ferrugineus* (Lesson, 1831)	RFA	Traumatogenic	VU	4.1	r1,5
Order Lamniformes					
Family Myliobatidae					
Genus *Aetobatus*					
*Aetobatus narinari* (Euphrasen, 1790)	BRA	Traumatogenic	NT	4.2	r2
Genus *Aetomylaeus*					
*Aetomylaeus maculatus* (Gray, 1834)	BRA	Traumatogenic	EN	3.8	r2
*Aetomylaeus vespertilio* (Bleeker, 1852)	BEP		EN	3.8	r2
Family Alopiidae					
Genus *Alopias*					
*Alopias vulpinus* (Bonnaterre, 1788)	PEL		VU	4.5	r1,r6
Family Lamnidae					
Genus *Isurus*					
*Isurus oxyrinchus* Rafinesque, 1810	PEL	Traumatogenic	NT	4.5	r2
Order Carcharhiniformes					
Family Scyliorhinidae					
Genus *Atelomycterus*					
*Atelomycterus marmoratus* (Anonymous [Bennett], 1830)	RFA		NT	4.1	r1
Genus *Cephaloscyllium*					
*Cephaloscyllium isabellum* (Bonnaterre, 1788)	RFA		LC	4.2	r2
*Cephaloscyllium umbratile* Jordan & Fowler, 1903	RFA			4.5	r4
Family Triakidae					
Genus *Mustelus*					
*Mustelus griseus* Pietschmann, 1908	DEM			3.5	r3
Family Carcharhinidae					
Genus *Carcharhinus*					
*Carcharhinus albimarginatus* (Rüppell, 1837)	RFA	Traumatogenic	VU	4.2	r1
*Carcharhinus amblyrhynchoides* (Whitley, 1934)	PN	Traumatogenic	NT	4.2	r1,r7
*Carcharhinus falciformis* (Müller & Henle, 1839)	RFA	Traumatogenic	VU	4.5	r1
*Carcharhinus limbatus* (Müller & Henle, 1839)	BRA	Traumatogenic	NT	4.4	r2
*Carcharhinus longimanus* (Poey, 1861)	PEL	Traumatogenic	VU	4.2	r1
Genus *Galeocerdo*					
*Galeocerdo cuvier* (Péron & Lesueur, 1822)	BRA	Traumatogenic	NT	4.5	r1
Genus *Glyphis*					
*Glyphis gangeticus* (Müller & Henle, 1839)	DEM; AMP	Traumatogenic	CE	4.2	r1
Genus *Hemigaleus*					
*Hemigaleus microstoma* Bleeker, 1852	DEM		VU	4.2	r4
Genus *Prionace*					
*Prionace glauca* (Linnaeus, 1758)	BRA	Traumatogenic	NT	4.4	r1
Genus *Rhizoprionodon*					
*Rhizoprionodon acutus* (Rüppell, 1837)	BEP; AMP		LC	4.3	r2
Genus *Scoliodon*					
*Scoliodon laticaudus* Müller & Henle, 1838	BRA		NT	3.8	r1,r7
Genus *Triaenodon*					
*Triaenodon obesus* (Rüppell, 1837)	RFA	Traumatogenic	NT	4.2	r1
Family Sphyrnidae					
Genus *Sphyrna*					
*Sphyrna lewini* (Griffith & Smith, 1834)	BRA	Other	VU	4.1	r1
Order Hexanchiformes					
Family Hexanchidae					
Genus *Hexanchus*					
*Hexanchus griseus* (Bonnaterre, 1788)	BAD	Poisonous to eat	NT	4.5	r1,r7
*Hexanchus nakamurai* Teng, 1962	BAD			4.2	r1
Genus *Notorynchus*					
*Notorynchus cepedianus* (Péron, 1807)	BRA	Traumatogenic	LR	4.7	r4
Genus *Myripristis*					
*Myripristis botche* Cuvier, 1829	RFA		LC	4.0	r1
*Myripristis chryseres* Jordan & Evermann, 1903	BEP		LC	4.0	r2
*Myripristis kuntee* Valenciennes, 1831	RFA		LC	3.4	r6,r8
*Myripristis murdjan* (Forsskål, 1775)	RFA	Reports of ciguatera poisoning	LC	3.4	r1,r7
*Myripristis pralinia* Cuvier, 1829	RFA		LC	3.5	r1
*Myripristis violacea* Bleeker, 1851	RFA		LC	3.5	r4
*Myripristis vittata* Valenciennes, 1831	RFA		LC	3.8	r3
*Myripristis robusta Randall & Greenfield, 1996	DEM				r11
Genus *Neoniphon*					
*Neoniphon argenteus* (Valenciennes, 1831)	RFA		LC	4.0	r3
*Neoniphon opercularis* (Valenciennes, 1831)	RFA	Venomous	LC	3.6	r1,r6
*Neoniphon sammara* (Forsskål, 1775)	RFA	Venomous	LC	3.6	r1,r6,r8,r11
Genus *Ostichthys*					
*Ostichthys kaianus* (Günther, 1880)	BAD		LC	4.0	r3
*Ostichthys sheni* Chen, Shao & Mok, 1990	DEM			3.7	r3
Genus *Sargocentron*					
*Sargocentron spiniferum* (Forsskål, 1775)	RFA	Reports of ciguatera poisoning	LC	3.6	r1
*Sargocentron violaceum* (Bleeker, 1853)	RFA	Venomous	LC	3.6	r1
*Sargocentron caudimaculatum* (Rüppell, 1838)	RFA	Venomous	LC	3.9	r4,r6,r11
*Sargocentron cornutum* (Bleeker, 1854)	BRA	Venomous	LC	3.6	r4
*Sargocentron diadema* (Lacepède, 1802)	RFA	Venomous	LC	3.4	r1
*Sargocentron ensifer* (Jordan & Evermann, 1903)	RFA		LC	4.0	r1,r7
*Sargocentron punctatissimum* (Cuvier, 1829)	RFA		LC	3.4	r1,r7
*Sargocentron rubrum* (Forsskål, 1775)	RFA	Venomous	LC	3.6	r4,r7
*Sargocentron spiniferum* (Forsskål, 1775)	RFA	Reports of ciguatera poisoning	LC	3.6	r4
*Sargocentron tiere* (Cuvier, 1829)	RFA	Venomous	LC	4.2	r3
*Sargocentron violaceum* (Bleeker, 1853)	RFA	Venomous	LC	3.6	r4
*Sargocentron melanospilos* (Bleeker, 1858)	RFA	Venomous	LC	4.0	r9,r11
*Sargocentron microstoma* (Günther, 1859)	RFA	Venomous	LC	3.6	r10,r11
Order Squatiniformes					
Family Squalidae					
Genus *Centrophorus*					
*Centrophorus tessellatus* Garman, 1906	BEP			4.3	r1
Genus *Squalus*					
*Squalus brevirostris* Tanaka, 1917	DEM			4.2	r1
*Squalus megalops* (Macleay, 1881)	DEM			4.3	r2
Order Rajiformes					
Family Rhinobatidae					
Genus *Rhina*					
*Rhina ancylostoma* Bloch & Schneider, 1801	RFA	Other	VU	3.6	r1
Genus *Rhynchobatus*					
*Rhynchobatus djiddensis* (Forsskål, 1775)	BRA		VU	3.6	r1
Order Myliobatiformes					
Family Dasyatidae					
Genus *Hemitrygon*					
*Hemitrygon bennettii* (Müller & Henle, 1841)	DEM			4.5	r2
*Hemitrygon sinensis* (Steindachner, 1892)	DEM			3.7	r2
Genus *Neotrygon*					
*Neotrygon kuhlii* (Müller & Henle, 1841)	RFA	Venomous		3.3	r2
Genus *Pteroplatytrygon*					
*Pteroplatytrygon violacea* (Bonaparte, 1832)	PEL	Venomous	LC	4.4	r1
Genus *Taeniurops*					
*Taeniurops meyeni* (Müller & Henle, 1841)	RFA	Venomous	VU	4.2	r1
Genus *Urogymnus*					
*Urogymnus asperrimus* (Bloch & Schneider, 1801)	BRA	Traumatogenic	VU	3.5	r2
Family Gymnuridae					
Genus *Gymnura*					
*Gymnura japonica* (Temminck & Schlegel, 1850)	DEM	Venomous		3.8	r2
Order Anguilliformes					
Family Muraenidae					
Genus *Echidna*					
*Echidna delicatula* (Kaup, 1856)	RFA			3.5	r1
*Echidna nebulosa* (Ahl, 1789)	RFA		LC	4.0	r1
*Echidna polyzona* (Richardson, 1845)	RFA		LC	3.5	r1
Genus *Gymnomuraena*					
*Gymnomuraena zebra* (Shaw, 1797)	RFA		LC	3.4	r1
Genus *Gymnothorax*					
*Gymnothorax fimbriatus* (Bennett, 1832)	BRA		LC	4.0	r1
*Gymnothorax flavimarginatus* (Rüppell, 1830)	RFA	Reports of ciguatera poisoning	LC	4.2	r1
*Gymnothorax isingteena* (Richardson, 1845)	RFA		LC	4.3	r4
*Gymnothorax meleagris* (Shaw, 1795)	RFA	Reports of ciguatera poisoning	LC	4.0	r1,r6,r11
*Gymnothorax pictus* (Ahl, 1789)	BRA	Reports of ciguatera poisoning	LC	4.2	r1
*Gymnothorax prionodon* Ogilby, 1895	RFA		LC	4.2	r3
*Gymnothorax pseudothyrsoideus* (Bleeker, 1853)	RFA		LC	3.7	r4
*Gymnothorax reevesii* (Richardson, 1845)	RFA			4.0	r1
*Gymnothorax richardsonii* (Bleeker, 1852)	RFA		LC	3.8	r1
*Gymnothorax rueppelliae* (McClelland, 1844)	BRA		LC	4.0	r1
*Gymnothorax thyrsoideus* (Richardson, 1845)	RFA		LC	4.0	r1
*Gymnothorax undulatus* (Lacepède, 1803)	BRA	Reports of ciguatera poisoning	LC	3.6	r1
Genus *Scuticaria*					
*Scuticaria tigrina* (Lesson, 1828)	RFA		LC	3.8	r1
Family Ophichthyidae					
Genus *Myrichthys*					
*Myrichthys colubrinus* (Boddaert, 1781)	RFA			3.6	r1
*Myrichthys maculosus* (Cuvier, 1816)	RFA			3.6	r1
Genus *Pisoodonophis*					
*Pisoodonophis rubicundus*				4.0	r1
Order Clupeiformes					
Family Clupeidae					
Genus *Amblygaster*					
*Amblygaster clupeoides* Bleeker, 1849	RFA		LC	3.1	r1
Genus *Conger*					
*Conger cinereus* Rüppell, 1830	RFA		LC	4.3	r1
Order Gonorhynchiformes					
Family Chanidae					
Genusa *Chanos*					
*Chanos chanos* (Forsskål, 1775)	BRA		LC	2.4	r1
Family Centriscidae					
Genus *Aeoliscus*					
*Aeoliscus strigatus* (Günther, 1861)	RFA			3.5	r6
Order Stomiiformes					
Family Sternoptychidae					
Genus *Sternoptyx*					
*Sternoptyx diaphana* Hermann, 1781	BAP			3.4	r1
*Sternoptyx obscura* Garman, 1899	BAP		LC	3.3	r1
Family Phosichthyidae					
Genus *Polymetme*					
*Polymetme corythaeola* (Alcock, 1898)	BEP			4.4	r1
Order Aulopiformes					
Family Synodontidae					
Genus *Saurida*					
*Saurida gracilis* (Quoy & Gaimard, 1824)	RFA		LC	4.2	r1
Genus *Synodus*					
*Synodus jaculum* Russell & Cressey, 1979	RFA		LC	4.0	r1
*Synodus variegatus* (Lacepède, 1803)	RFA		LC	4.2	r1
Family Alepisauridae					
Genus *Alepisaurus*					
*Alepisaurus ferox* Lowe, 1833	BAP		LC	4.0	r1
Order Myctophiformes					
Family Myctophidae					
Genus *Myctophum*					
*Myctophum aurolaternatum* Garman, 1899	BAP		LC	3.5	r1
Order Polymixiiformes					
Family Polymixiidae					
Genus *Polymixia*					
*Polymixia berndti* Gilbert, 1905	RFA		LC	4.0	r1
Order Ophidiiformes					
Family Carapidae					
Genus *Carapus*					
*Carapus mourlani* (Petit, 1934)	RFA			3.6	r1
Genus *Encheliophis*					
*Encheliophis homei* (Richardson, 1846)	DEM			3.7	r1
*Encheliophis boraborensis* (Kaup, 1856)	DEM			3.4	r1
Family Bythitidae					
Genus *Dinematichthys*					
*Dinematichthys iluocoeteoides* Bleeker, 1855	RFA			2.6	r1
Order Lophiiformes					
Family Antennaridae					
Genus *Antennatus*					
*Antennatus dorehensis* (Bleeker, 1859)	RFA			4.3	r1
Family Chaunacidae					
Genus *Chaunax*					
*Chaunax fimbriatus* Hilgendorf, 1879	BAD			3.9	r1
Family Ogcocephalidae					
Genus *Halicmetus*					
*Halicmetus reticulatus* Smith & Radcliffe, 1912	BAD			3.3	r1
Genus *Halieutaea*					
*Halieutaea indica* Annandale & Jenkins, 1910	DEM			3.4	r1
Order Mugiliformes					
Family Mugilidae					
Genus *Crenimugil*					
*Crenimugil crenilabis* (Forsskål, 1775)	BRA		LC	2.3	r1
Genus *Ellochelon*					
*Ellochelon vaigiensis* (Quoy & Gaimard, 1825)	RFA;		LC	2.2	r1
Genus *Moolgarda*					
*Crenimugil seheli* (Forsskål, 1775)	RFA;			2.3	r1
Genus *Oedalechilus*					
*Plicomugil labiosus* (Valenciennes, 1836)	RFA			2.1	r1
Order Atheriniformes					
Family Atherinidae					
Genus *Atherinomorus*					
*Atherinomorus lacunosus* (Forster, 1801)	RFA;			3.3	r1
Family Exocoetidae					
Genus *Cheilopogon*					
*Cheilopogon arcticeps* (Günther, 1866)	PN			3.6	r1
*Cheilopogon atrisignis* (Jenkins, 1903)	PEL			3.7	r1
*Cheilopogon cyanopterus* (Valenciennes, 1847)	PEL		LC	3.3	r1
*Cheilopogon katoptron* (Bleeker, 1865)	PN			3.5	r1
*Cheilopogon pinnatibarbatus*pinnatibarbatus (Bennett, 1831)	PN		LC	4.0	r1
*Cheilopogon spilopterus* (Valenciennes, 1847)	PN			4.2	r1
Genus *Cypselurus*					
*Cypselurus oligolepis* (Bleeker, 1865)	PN			4.0	r1
*Cypselurus poecilopterus* (Valenciennes, 1847)	PN; OD			3.4	r1
Genus *Exocoetus*					
*Exocoetus volitans* Linnaeus, 1758	PN; OD		LC	3.0	r1,r6
Genus *Hirundichthys*					
*Hirundichthys oxycephalus* (Bleeker, 1853)	PN; OD			3.0	r1
*Hirundichthys speculiger* (Valenciennes, 1847)	PEL; OD		LC	3.0	r1
Genus *Parexocoetus*					
*Parexocoetus brachypterus* (Richardson, 1846)	PN; OD			3.4	r1
Family Hemiramphidae					
Genus *Euleptorhamphus*					
*Euleptorhamphus viridis* (van Hasselt, 1823)	PEL; OD			3.4	r1,r8
Genus *Hyporhamphus*					
*Hyporhamphus dussumieri* (Valenciennes, 1847)	RFA			3.5	r1
*Hyporhamphus gernaerti* (Valenciennes, 1847)	PN			3.0	r1
Family Belonidae					
Genus *Ablennes*					
*Ablennes hians* (Valenciennes, 1846)	RFA; OD		LC	4.5	r1
Genus *Platybelone*					
*Platybelone argalus argalus* (Lesueur, 1821)	RFA		LC	4.5	r1
Genus *Tylosurus*					
*Tylosurus acus acus* (Lacepède, 1803)	RFA	Traumatogenic	LC	4.5	r1
Order Gasterosteiformes					
Family Pegasidae					
Genus *Pegasus*					
*Pegasus laternarius* Cuvier, 1816	DEM			3.3	r1
Family Aulostomidae					
Genus *Aulostomus*					
*Aulostomus chinensis* (Linnaeus, 1766)	RFA		LC	4.2	r1
Genus *Centriscus*					
*Centriscus scutatus* Linnaeus, 1758	RFA		LC	3.3	r1
Family Fistulariidae					
Genus *Fistularia*					
*Fistularia petimba* Lacepède, 1803	RFA		LC	4.4	r1,r8
Order synbranchiformes					
Family Syngnathidae					
Genus *Corythoichthys*					
*Corythoichthys flavofasciatus* (Rüppell, 1838)	RFA		LC	3.6	r1,r6
Genus *Doryrhamphus*					
*Doryrhamphus excisus excisus* Kaup, 1856	RFA		LC	3.5	r1
Genus *Trachyrhamphus*					
*Trachyrhamphus serratus* (Temminck & Schlegel, 1850)	RFA			3.7	r1
Order Scorpaeniformes					
Family Dactylopteridae					
Genus *Dactyloptena*					
*Dactyloptena orientalis* (Cuvier, 1829)	RFA		LC	3.7	r1
Family Scorpaenidae					
Genus *Dendrochirus*					
*Dendrochirus bellus* (Jordan & Hubbs, 1925)	DEM		LC	3.8	r1
*Dendrochirus zebra* (Cuvier, 1829)	RFA	Venomous	LC	4.0	r1
Genus *Parapterois*					
*Parapterois heterura* (Bleeker, 1856)	DEM	Venomous	LC	3.9	r1
Genus *Parascorpaena*					
*Parascorpaena aurita* (Rüppell, 1838)	RFA		LC	3.7	r1
*Parascorpaena picta* (Cuvier, 1829)	RFA		LC	3.7	r1
Genus *Pterois*					
*Pterois radiata* Cuvier, 1829	RFA	Venomous	LC	3.6	r1,r6
*Pterois volitans* (Linnaeus, 1758)	RFA	Venomous	LC	4.4	r1,r6
Genus *Scorpaena*					
*Scorpaena hatizyoensis* Matsubara, 1943	DEM		LC	3.7	r1
*Scorpaena neglecta* Temminck & Schlegel, 1843	DEM			3.8	r1
Genus *Scorpaenodes*					
*Scorpaenodes guamensis* (Quoy & Gaimard, 1824)	RFA	Venomous	LC	3.4	r1
*Scorpaenodes scaber* (Ramsay & Ogilby, 1886)	RFA	Venomous		3.6	r1
Genus *Scorpaenopsis*					
*Scorpaenopsis cirrosa* (Thunberg,1793)	DEM	Venomous		3.5	r1
Genus *Sebastapistes*					
*Sebastapistes cyanostigma (Bleeker, 1856)	RFA			3.8	r11
*Sebastapistes nuchalis* (Günther, 1874)	DEM			3.8	r1
Genus *Synanceia*					
*Synanceia verrucosa* Bloch & Schneider, 1801	RFA	Venomous	LC	4.2	r1
Genus *Vespicula*					
*Vespicula trachinoides* (Cuvier, 1829)	DEM			3.1	r1
Order perciformes					
Family Serranidae					
Genus *Aethaloperca*					
*Aethaloperca rogaa* (Forsskål, 1775)	RFA		LC	4.2	r1,r11
Genus *Anyperodon*					
*Anyperodon leucogrammicus* (Valenciennes, 1828)	RFA		LC	3.9	r1
Genus *Cephalopholis*					
*Cephalopholis argus* Schneider, 1801	RFA	Reports of ciguatera poisoning	LC	4.5	r1,r6,r11
*Cephalopholis aurantia* (Valenciennes, 1828)	RFA		LC	4.0	r1
*Cephalopholis formosa* (Shaw, 1812)	RFA		LC	4.1	r1
*Cephalopholis leopardus* (Lacepède, 1801)	RFA		LC	4.0	r1
*Cephalopholis miniata* (Forsskål, 1775)	RFA		LC	4.3	r1
*Cephalopholis sonnerati* (Valenciennes, 1828)	RFA		LC	3.8	r1
*Cephalopholis urodeta* (Forster, 1801)	RFA		LC	4.0	r1,r6,r11
Genus *Epinephelus*					
*Epinephelus akaara* (Temminck & Schlegel, 1842)	RFA		EN	4.0	r1
*Epinephelus areolatus* (Forsskål, 1775)	RFA		LC	3.7	r1
*Epinephelus awoara* (Temminck & Schlegel, 1842)	RFA			3.6	r1
*Epinephelus chlorostigma* (Valenciennes, 1828)	RFA		LC	4.0	r1
*Epinephelus coioides* (Hamilton, 1822)	RFA		LC	4.0	r1
*Epinephelus cyanopodus* (Richardson, 1846)	RFA	Reports of ciguatera poisoning	LC	4.1	r4,r6
*Epinephelus fasciatus* (Forsskål, 1775)	RFA	Reports of ciguatera poisoning	LC	3.7	r1,r7,r11
*Epinephelus fuscoguttatus* (Forsskål, 1775)	RFA	Reports of ciguatera poisoning	VU	4.1	r1
*Epinephelus heniochus* Fowler, 1904	DEM		LC	3.8	r1
*Epinephelus hexagonatus* (Forster, 1801)	RFA		LC	4.1	r1,r11
*Epinephelus latifasciatus* (Temminck & Schlegel, 1842)	DEM		LC	4.0	r1
*Epinephelus lanceolatus* (Bloch, 1790)	RFA	Traumatogenic		4.0	r1
*Epinephelus longispinis* (Kner, 1864)	RFA		LC	4.2	r1
*Epinephelus maculatus* (Bloch, 1790)	RFA	Reports of ciguatera poisoning	LC	4.0	r5
*Epinephelus merra* Bloch, 1793	RFA	Reports of ciguatera poisoning	LC	3.8	r1,r6,r8,r11
*Epinephelus morrhua* (Valenciennes, 1833)	RFA	Reports of ciguatera poisoning	LC	4.0	r1
*Epinephelus poecilonotus* (Temminck & Schlegel, 1842)	RFA		LC	4.0	r1
*Epinephelus retouti* Bleeker, 1868	RFA		LC	4.0	r1
*Epinephelus spilotoceps* Schultz, 1953	RFA		LC	3.7	r1,r11
*Epinephelus summana* (Forsskål, 1775)	RFA		LC	3.8	r1
*Epinephelus tauvina* (Forsskål, 1775)	RFA; OD	Reports of ciguatera poisoning		4.1	r1
Genus *Gracila*					
*Gracila albomarginata* (Fowler & Bean, 1930)	RFA		LC	4.2	r1
Genus *Grammistes*					
*Grammistes sexlineatus* (Thunberg, 1792)	RFA	Reports of ciguatera poisoning	LC	4.0	r1,r11
Genus *Plectropomus*					
*Plectropomus areolatus* (Rüppell, 1830)	RFA	Reports of ciguatera poisoning	VU	4.5	r1
*Plectropomus laevis* (Lacepède, 1801)	RFA	Reports of ciguatera poisoning	LC	4.1	r1
*Plectropomus leopardus* (Lacepède, 1802)	RFA	Reports of ciguatera poisoning	LC	4.4	r1
*Plectropomus oligacanthus* (Bleeker, 1855)	RFA	Reports of ciguatera poisoning	LC	4.0	r1,r7
Genus *Pogonoperca*					
*Pogonoperca ocellata* Günther, 1859	RFA		LC	4.0	r1
Genus *Pseudanthias*					
*Pseudanthias cichlops* (Bleeker, 1853)	RFA		LC	3.4	r5
*Pseudanthias pascalus* (Jordan & Tanaka, 1927)	RFA		LC	3.3	r6,r11
*Pseudanthias tuka* (Herre & Montalban, 1927)	RFA		LC	3.6	r1
Genus *Saloptia*					
*Saloptia powelli* Smith, 1964	RFA		LC	3.9	r1
Genus *Variola*					
*Variola louti* (Forsskål, 1775)	RFA	Reports of ciguatera poisoning	LC	4.3	r1
Family Pseudochromidae					
Genus *Labracinus*					
*Labracinus cyclophthalmus* (Müller & Troschel, 1849)	RFA			3.9	r1
Genus *Pseudochromis*					
*Pseudochromis fuscus* Müller & Troschel, 1849	RFA		LC	3.5	r1
Family Plesiopida					
Genus *Plesiops*					
*Plesiops coeruleolineatus* Rüppell, 1835	RFA			3.6	r1
Family Priacanthidae					
Genus *Heteropriacanthus*					
*Heteropriacanthus cruentatus* (Lacepède, 1801)	RFA	Reports of ciguatera poisoning	LC	3.6	r1
Genus *Priacanthus*					
*Priacanthus hamrur* (Forsskål, 1775)	RFA		LC	3.6	r1,r7
*Priacanthus macracanthus* Cuvier, 1829	RFA; OD		LC	4.1	r6
Family Apogonidae					
Genus *Cheilodipterus*					
*Cheilodipterus quinquelineatus* Cuvier, 1828	RFA			3.9	r1,r8,r11
Genus *Fowleria*					
*Fowleria aurita* (Valenciennes, 1831)	RFA			3.5	r1
Genus *Nectamia*					
*Nectamia bandanensis* (Bleeker, 1854)	RFA			3.3	r1
Genus *Ostorhinchus*					
*Ostorhinchus nigrofasciatus* (Lachner, 1953)	RFA			3.6	r8,r11
*Ostorhinchus novemfasciatus* (Cuvier, 1828)	RFA			4.0	r1,r11
*Ostorhinchus cookii* (Macleay, 1881)	RFA			4.0	r6,r11
Genus *Pristicon*					
*Pristicon trimaculatus* (Cuvier, 1828)	RFA			3.5	r1
Genus *Sphaeramia*					
*Sphaeramia orbicularis* (Cuvier, 1828)	RFA			3.6	r1
Family Malacanthidae					
Genus *Malacanthus*					
*Malacanthus brevirostris* Guichenot, 1848	RFA			3.5	r1
*Malacanthus latovittatus* (Lacepède, 1801)	RFA			3.5	r1
Family Microdesmidae					
Genus *Ptereleotris*					
*Ptereleotris microlepis* (Bleeker, 1856)	RFA			3.4	r1
Family Coryphaenidae					
Genus *Coryphaena*					
*Coryphaena hippurus* Linnaeus, 1758	PN; OD	Reports of ciguatera poisoning	LC	4.4	r1
Family Echeneidae					
Genus *Echeneis*					
*Echeneis naucrates* Linnaeus, 1758	RFA		LC	3.7	r1
Genus *Remora*					
*Remora albescens* (Temminck & Schlegel, 1850)	PEL; OD		LC	3.4	r1
*Remora brachyptera* (Lowe, 1839)	PEL; OD		LC	3.5	r1
*Remora remora* (Linnaeus, 1758)	RFA		LC	3.5	r1
Genus *Rhombochirus*					
*Remora osteochir* (Cuvier, 1829)	RFA		LC	3.5	r1
Family Carangidae					
Genus *Carangoides*					
*Carangoides ferdau* (Forsskål, 1775)	RFA	Reports of ciguatera poisoning	LC	4.3	r1
Genus *Caranx*					
*Caranx ignobilis* (Forsskål, 1775)	RFA	Reports of ciguatera poisoning	LC	4.2	r1,r7
*Caranx melampygus* Cuvier, 1833	RFA	Reports of ciguatera poisoning	LC	4.5	r1,r11
*Caranx sexfasciatus* Quoy & Gaimard, 1825	RFA		LC	4.5	r1
Genus *Decapterus*					
*Decapterus macrosoma* Bleeker, 1851	RFA		LC	3.4	r1,r7
Genus *Elagatis*					
*Elagatis bipinnulata* (Quoy & Gaimard, 1825)	RFA	Reports of ciguatera poisoning	LC	4.3	r1
Genus *Naucrates*					
*Naucrates ductor* (Linnaeus, 1758)	RFA		LC	3.4	r1
Genus *Scomberoides*					
*Scomberoides lysan* (Forsskål, 1775)	RFA	Other	LC	4.0	r1
Genus *Selar*					
*Selar crumenophthalmus* (Bloch, 1793)	RFA	Reports of ciguatera poisoning	LC	3.8	r1,r7
Genus *Selaroides*					
*Selaroides leptolepis* (Cuvier, 1833)	RFA; AMP		LC	3.8	r1
Genus *Seriola*					
*Seriola dumerili* (Risso, 1810)	RFA; OD	Reports of ciguatera poisoning	LC	4.5	r1
*Seriola lalandi* Valenciennes, 1833	BEP		LC	4.2	r1
*Seriola quinqueradiata* Temminck & Schlegel, 1845	DEM; OD		LC	4.0	r1
Genus *Trachinotus*					
*Trachinotus baillonii* (Lacepède, 1801)	RFA		LC	3.6	r1
Genus *Alectis*					
*Alectis indica* (Rüppell, 1830)	RFA		LC	4.1	r1
Genus *Caranx*					
*Caranx lugubris* Poey, 1860	BEP; OD	Reports of ciguatera poisoning	LC	4.5	r1
Family Bramidae					
Genus *Brama*					
*Brama japonica* Hilgendorf, 1878	PEL; OD			4.4	r1
Family Lutjanidae					
Genus *Aphareus*					
*Aphareus furca* (Lacepède, 1801)	RFA	Reports of ciguatera poisoning	LC	4.1	r1
*Aphareus rutilans* Cuvier, 1830	RFA		LC	4.1	r1
Genus *Aprion*					
*Aprion virescens* Valenciennes, 1830	RFA	Reports of ciguatera poisoning	LC	4.3	r1,r6,r11
Genus *Etelis*					
*Etelis carbunculus* Cuvier, 1828	BEP		LC	4.5	r1
Genus *Lutjanus*					
*Lutjanus argentimaculatus* (Forsskål, 1775)	RFA; OD	Reports of ciguatera poisoning	LC	3.6	r1
*Lutjanus bohar* (Forsskål, 1775)	RFA	Reports of ciguatera poisoning	LC	4.3	r1
*Lutjanus erythropterus* Bloch, 1790	RFA		LC	4.5	r1
*Lutjanus fulviflamma* (Forsskål, 1775)	RFA	Reports of ciguatera poisoning	LC	3.8	r1
*Lutjanus fulvus* (Forster, 1801)	RFA	Reports of ciguatera poisoning	LC	3.6	r1,r11
*Lutjanus gibbus* (Forsskål, 1775)	RFA	Reports of ciguatera poisoning	LC	4.1	r1,r11
*Lutjanus kasmira* (Forsskål, 1775)	RFA		LC	3.9	r1,r7
*Lutjanus monostigma* (Cuvier, 1828)	RFA	Reports of ciguatera poisoning	LC	4.3	r1
*Lutjanus quinquelineatus* (Bloch, 1790)	RFA		LC	3.7	r1
*Lutjanus russellii* (Bleeker, 1849)	RFA		LC	4.1	r1
*Lutjanus sebae* (Cuvier, 1816)	RFA	Reports of ciguatera poisoning	LC	4.1	r1
*Lutjanus stellatus* Akazaki, 1983	RFA			4.0	r1
Genus *Macolor*					
*Macolor niger* (Forsskål, 1775)	RFA		LC	4.0	r1
Genus *Paracaesio*					
*Paracaesio sordida* Abe & Shinohara, 1962	RFA		LC	2.8	r1
*Paracaesio xanthura* (Bleeker, 1869)	RFA		LC	3.4	r1
Genus *Pristipomoides*					
*Pristipomoides argyrogrammicus* (Valenciennes, 1832)	RFA		LC	4.2	r1
*Pristipomoides auricilla* (Jordan, Evermann & Tanaka, 1927)	BEP		LC	3.9	r1
*Pristipomoides filamentosus* (Valenciennes, 1830)	BEP		LC	4.2	r1
*Pristipomoides flavipinnis* Shinohara, 1963	RFA		LC	3.6	r1
*Pristipomoides multidens* (Day, 1871)	DEM		LC	3.8	r1,r7
*Pristipomoides sieboldii* (Bleeker, 1855)	BEP		LC	3.7	r5
Genus *Symphorichthys*					
*Symphorichthys spilurus* (Günther, 1874)	RFA		LC	3.8	r1
Family Caesionidae					
Genus *Caesio*					
*Caesio caerulaurea* Lacepède, 1801	RFA		LC	3.4	r1,r11
*Caesio lunaris* Cuvier, 1830	RFA		LC	3.4	r1,r7
*Caesio teres* Seale, 1906	RFA		LC	3.4	r6
*Caesio xanthonota* Bleeker, 1853	RFA		LC	3.4	r1
Genus *Pterocaesio*					
*Pterocaesio digramma* (Bleeker, 1864)	RFA		LC	3.4	r1,r6,r7
*Pterocaesio tile* (Cuvier, 1830)	RFA		LC	3.3	r1,r6,r11
*Pterocaesio trilineata Carpenter, 1987	RFA		LC	3.4	r10,r11
Family Gerridae					
Genus *Gerres*					
*Gerres filamentosus* Cuvier, 1829	DEM; AMP		LC	3.3	r1
*Gerres longirostris* (Lacepède, 1801)	RFA		LC	3.5	r1
*Gerres oblongus* Cuvier, 1830	RFA		LC	3.5	r1
*Gerres oyena* (Forsskål, 1775)	RFA		LC	2.7	r1
Family Callionymidae					
Genus *Neosynchiropus*					
*Neosynchiropus ocellatus* (Pallas, 1770)	RFA			3.2	r1
Family Haemulidae					
Genus *Diagramma*					
*Diagramma pictum* (Thunberg, 1792)	RFA	Reports of ciguatera poisoning		3.7	r1
Genus *Plectorhinchus*					
*Plectorhinchus chaetodonoides* Lacepède, 1801	RFA			3.8	r1,r8,r11
*Plectorhinchus diagrammus* (Linnaeus, 1758)	RFA			4.0	r1
*Plectorhinchus flavomaculatus* (Cuvier, 1830)	RFA			4.0	r1
*Plectorhinchus lineatus* (Linnaeus, 1758)	RFA			3.9	r1
*Plectorhinchus picus* (Cuvier, 1828)	RFA			3.9	r1
*Plectorhinchus vittatus* (Linnaeus, 1758)	RFA		LC	3.9	r1,r11
Family Nemipteridae					
Genus *Pentapodus*					
*Pentapodus caninus* (Cuvier, 1830)	RFA		LC	3.6	r1,r11
*Pentapodus nagasakiensis* (Tanaka, 1915)	RFA		LC	3.4	r1
Genus *Scolopsis*					
*Scolopsis aurata* (Park, 1797)	RFA		LC	3.6	r1
*Scolopsis bilineata* (Bloch, 1793)	RFA		LC	3.6	r1,r11
*Scolopsis lineata* Quoy & Gaimard, 1824	RFA		LC	3.8	r1,r8,r11
*Scolopsis monogramma* (Cuvier, 1830)	RFA		LC	3.5	r6
*Scolopsis taenioptera* (Cuvier, 1830)	DEM		LC	3.9	r1
Family Lethrinidae					
Genus *Gnathodentex*					
*Gnathodentex aureolineatus* (Lacepède, 1802)	RFA	Reports of ciguatera poisoning	LC	3.7	r1,r6,r7,r8
Genus *Gymnocranius*					
*Gymnocranius griseus* (Temminck & Schlegel, 1843)	RFA		LC	3.2	r1,r7
Genus *Lethrinus*					
*Lethrinus haematopterus* Temminck & Schlegel, 1844	RFA			3.7	r1
*Lethrinus lentjan* (Lacepède, 1802)	RFA		LC	3.9	r1,r7
*Lethrinus miniatus* (Forster, 1801)	RFA	Reports of ciguatera poisoning	LC	4.2	r1
*Lethrinus nebulosus* (Forsskål, 1775)	RFA	Reports of ciguatera poisoning	LC	3.8	r1,r6
*Lethrinus ornatus* Valenciennes, 1830	RFA		LC	3.4	r1
*Lethrinus rubrioperculatus* Sato, 1978	RFA		LC	3.8	r1,r11
*Lethrinus variegatus* Valenciennes, 1830	RFA		LC	3.8	r1,r7
*Lethrinus xanthochilus* Klunzinger, 1870	RFA		LC	3.8	r1
Genus *Monotaxis*					
*Monotaxis grandoculis* (Forsskål, 1775)	RFA	Reports of ciguatera poisoning	LC	3.4	r1,r8,r11
Genus *Pentapodus*					
*Pentapodus setosus* (Valenciennes, 1830)	RFA			3.5	r1
Family Sparidae					
Genus *Dentex*					
*Dentex tumifrons* (Temminck & Schlegel, 1843)	DEM		LC	3.8	r1
Family Mullidae					
Genus *Mulloidichthys*					
*Mulloidichthys flavolineatus* (Lacepède, 1801)	RFA		LC	3.8	r1
*Mulloidichthys vanicolensis* (Valenciennes, 1831)	RFA		LC	3.6	r1,r6
Genus *Parupeneus*					
*Parupeneus barberinus* (Lacepède, 1801)	RFA		LC	3.4	r1,r6,r7,r8,r11
*Parupeneus ciliatus* (Lacepède, 1802)	RFA		LC	3.5	r1,r6
*Parupeneus crassilabris* (Valenciennes, 1831)	RFA		LC	3.6	r6
*Parupeneus cyclostomus* (Lacepède, 1801)	RFA	Reports of ciguatera poisoning	LC	4.2	r1,r6,r8,r11
*Parupeneus forsskali* (Fourmanoir & Guézé, 1976)	RFA			3.5	r4,r6
*Parupeneus multifasciatus* (Quoy & Gaimard, 1825)	RFA		LC	3.5	r6,r8,r11
*Parupeneus pleurostigma* (Bennett, 1831)	RFA		LC	3.4	r1,r6
*Parupeneus trifasciatus* (Lacepède, 1801)	RFA			3.5	r1,r11
*Parupeneus heptacanthus* (Lacepède, 1802)	RFA		LC	3.4	r7
Genus *Upeneus*					
*Upeneus subvittatus* (Temminck & Schlegel, 1843)	DEM			4.2	r1
Family Pempheridae					
Genus *Pempheris*					
*Pempheris oualensis* Cuvier, 1831	RFA			3.6	r2,r6
Family Kyphosidae					
Genus *Kyphosus*					
*Kyphosus cinerascens* (Forsskål, 1775)	RFA	Poisonous to eat	LC	2.9	r1
*Kyphosus vaigiensis* (Quoy & Gaimard, 1825)	RFA; OD		LC	2.0	r1
Family Chaetodontidae					
Genus *Chaetodon*					
*Chaetodon adiergastos* Seale, 1910	RFA		LC	3.5	r1
*Chaetodon auriga* Forsskål, 1775	RFA		LC	3.7	r1,r8,r11
*Chaetodon auripes* Jordan & Snyder, 1901	RFA		LC	3.5	r6,r11
*Chaetodon bennetti* Cuvier, 1831	RFA			3.1	r1,9
*Chaetodon citrinellus* Cuvier, 1831	RFA		LC	3.5	r1,r11
*Chaetodon collare* Bloch, 1787	RFA		LC	3.4	r1
*Chaetodon ephippium* Cuvier, 1831	RFA		LC	3.0	r1,r8
*Chaetodon falcula* Bloch, 1795	RFA		LC	3.5	r1
*Chaetodon guttatissimus* Bennett, 1833	RFA		LC	3.1	r1
*Chaetodon kleinii* Bloch, 1790	RFA		LC	2.9	r1,r11
*Chaetodon lineolatus* Cuvier, 1831	RFA		LC	3.4	r1.r11
*Chaetodon lunula* (Lacepède, 1802)	RFA		LC	3.7	r1,r6,r8
*Chaetodon madagaskariensis* Ahl, 1923	RFA		LC	2.8	r1,r6,r11
*Chaetodon melannotus* Bloch & Schneider, 1801	RFA		LC	4.4	r1,r6,r8
*Chaetodon ornatissimus* Cuvier, 1831	RFA		LC	3.3	r1,r6,r11
*Chaetodon punctatofasciatus* Cuvier, 1831	RFA		LC	3.4	r1,r6,r11
*Chaetodon rafflesii Anonymous* [Bennett], 1830	RFA		LC	4.3	r2,r6,r11
*Chaetodon semeion* Bleeker, 1855	RFA		LC	2.7	r1
*Chaetodon speculum* Cuvier, 1831	RFA		LC	3.6	r1,r11
*Chaetodon trifascialis* Quoy & Gaimard, 1825	RFA		NT	3.3	r1,r6,r11
*Chaetodon trifasciatus* Park, 1797	RFA		LC	3.3	r1
*Chaetodon ulietensis* Cuvier, 1831	RFA		LC	2.7	r6
*Chaetodon unimaculatus* Bloch, 1787	RFA		LC	3.3	r1,r6
*Chaetodon vagabundus* Linnaeus, 1758	RFA		LC	2.9	r1,r6,r11
*Chaetodon wiebeli* Kaup, 1863	RFA		LC	2.7	r1,r6
*Chaetodon xanthurus* Bleeker, 1857	RFA		LC	2.8	r6,r7,r8,r11
*Chaetodon lunulatus* Quoy & Gaimard, 1825	RFA		LC	3.3	r10,r11
Genus *Coradion*					
*Coradion chrysozonus* (Cuvier, 1831)	RFA		LC	2.8	r1
Genus *Forcipiger*					
*Forcipiger flavissimus* Jordan & McGregor, 1898	RFA		LC	3.1	r6,r11
*Forcipiger longirostris* (Broussonet, 1782)	RFA		LC	3.5	r1,r11
Genus *Hemitaurichthys*					
*Hemitaurichthys polylepis* (Bleeker, 1857)	RFA		LC	3.1	r6,r11
*Hemitaurichthys zoster* (Bennett, 1831)	RFA		LC	3.3	r1
Genus *Heniochus*					
*Heniochus acuminatus* (Linnaeus, 1758)	RFA		LC	3.5	r1
*Heniochus chrysostomus* Cuvier, 1831	RFA		LC	3.8	r1,r8,r11
*Heniochus monoceros* Cuvier, 1831	RFA		LC	3.5	r1
*Heniochus singularius* Smith & Radcliffe, 1911	RFA		LC	3.6	r1
*Heniochus varius* (Cuvier, 1829)	RFA		LC	3.2	r1
Genus *Roa*					
*Roa modesta* (Temminck & Schlegel, 1844)	RFA; OD		LC	3.5	r1
Genus *Parachaetodon*					
*Parachaetodon ocellatus* (Cuvier, 1831)	RFA		LC	2.8	r1
Family Pomacanthidae					
Genus *Apolemichthys*					
*Apolemichthys trimaculatus* (Cuvier, 1831)	RFA		LC	2.6	r1,r11
Genus *Centropyge*					
*Centropyge bispinosa* (Günther, 1860)	RFA		LC	2.8	r1,r6,r8,r11
*Centropyge heraldi* Woods & Schultz, 1953	RFA		LC	2.8	r1,r11
*Centropyge vrolikii* (Bleeker, 1853)	RFA		LC	2.8	r1,r6
*Centropyge tibicen (Cuvier, 1831)	RFA		LC	2.8	r11
Genus *Genicanthus*					
*Genicanthus melanospilos* (Bleeker, 1857)	RFA		LC	3.4	r1
Genus *Pomacanthus*					
*Pomacanthus annularis* (Bloch, 1787)	RFA		LC	2.6	r1
*Pomacanthus imperator* (Bloch, 1787)	RFA		LC	2.7	r1,r6,r11
*Pomacanthus semicirculatus* (Cuvier, 1831)	RFA		LC	2.7	r1
*Pomacanthus sexstriatus* (Cuvier, 1831)	RFA		LC	2.6	r1,r6
Genus *Pygoplites*					
*Pygoplites diacanthus* (Boddaert, 1772)	RFA		LC	2.7	r1,r6,r11
Family Pentacerotidae					
Genus *Histiopterus*					
*Histiopterus typus* Temminck & Schlegel, 1844	RFA			3.5	r1
Family Theraponidae					
Genus *Therapon*					
*Terapon jarbua* (Forsskål, 1775)	DEM		LC	3.9	r1
*Terapon theraps* Cuvier, 1829	RFA		LC	3.5	r1
Family Kuhliidae					
Genus *Kuhlia*					
*Kuhlia mugil* (Forster, 1801)	RFA		LC	3.8	r1
Family Cirrhitidae					
Genus *Cirrhitichthys*					
*Cirrhitichthys aureus* (Temminck & Schlegel, 1842)	RFA			4.0	r1
*Cirrhitichthys falco* Randall, 1963	RFA		LC	4.0	r6,r11
*Cirrhitichthys oxycephalus* (Bleeker, 1855)	RFA		LC	4.0	r9,r10,r11
Genus *Cirrhitus*					
*Cirrhitus pinnulatus* (Forster, 1801)	RFA		LC	3.7	r1,r11
Genus *Paracirrhites*					
*Paracirrhites arcatus* (Cuvier, 1829)	RFA		LC	3.6	r1,r6,r11
Family Pomacentridae					
Genus *Abudefduf*					
*Abudefduf septemfasciatus* (Cuvier, 1830)	RFA		LC	3.0	r1
*Abudefduf sexfasciatus* (Lacepède, 1801)	RFA		LC	2.7	r1,r11
*Abudefduf sordidus* (Forsskål, 1775)	RFA		LC	2.9	r1,r6,r7
*Abudefduf vaigiensis* (Quoy & Gaimard, 1825)	RFA	Reports of ciguatera poisoning	LC	2.6	r1,r6,r8,r11
*Abudefduf bengalensis (Bloch, 1787)	RFA		LC	3.1	r11
Genus *Amblyglyphidodon*					
*Amblyglyphidodon aureus* (Cuvier, 1830)	RFA		LC	2.7	r1
*Amblyglyphidodon leucogaster* (Bleeker, 1847)	RFA		LC	3.4	r1,r6,r11
*Amblyglyphidodon curacao* (Bloch, 1787)	RFA		LC	2.6	r8,r11
Genus *Amphiprion*					
*Amphiprion akallopisos* Bleeker, 1853	RFA		LC	2.7	r1
*Amphiprion bicinctus* Rüppell, 1830	RFA		LC	2.7	r1
*Amphiprion clarkii* (Bennett, 1830)	RFA			2.9	r6,r11
*Amphiprion frenatus* Brevoort, 1856	RFA		LC	2.7	r1,r6,r11
*Amphiprion perideraion* Bleeker, 1855	RFA		LC	2.2	r1,r6
*Amphiprion percula (Lacepède, 1802)	RFA		LC	2.7	r11
Genus *Chromis*					
*Chromis chrysura* (Bliss, 1883)	RFA			3.0	r1
*Chromis dimidiata* (Klunzinger, 1871)	RFA		LC	2.7	r1
*Chromis margaritifer* Fowler, 1946	RFA			3.0	r6,r11
*Chromis notata* (Temminck & Schlegel, 1843)	RFA			3.4	r1
*Chromis ternatensis* (Bleeker, 1856)	RFA			3.4	r1,r6
*Chromis xanthura* (Bleeker, 1854)	RFA			3.4	r1,r11
*Chromis fumea (Tanaka, 1917)	RFA		LC	3.4	r11
*Chromis lepidolepis Bleeker, 1877	RFA			3.4	r11
*Chromis ovatiformes* Fowler, 1946	RFA			3.4	r9,r10,r11
*Chromis caudalis Randall, 1988	RFA		LC	3.0	r11
*Chromis viridis (Cuvier, 1830)	RFA			2.9	r9,r11
Genus *Chrysiptera*					
*Chrysiptera biocellata* (Quoy & Gaimard, 1825)	RFA			2.0	r1,r8,r11
*Chrysiptera brownriggii* (Bennett, 1828)	RFA			2.7	r1
*Chrysiptera cyanea* (Quoy & Gaimard, 1825)	RFA			2.5	r1,r6
*Chrysiptera glauca* (Cuvier, 1830)	RFA			2.4	r1
*Chrysiptera chrysocephala* Manica, Pilcher & Oakley, 2002	PN			2.7	r9,r11
*Chrysiptera unimaculata (Cuvier, 1830)	RFA		LC	2.1	r11
*Chrysiptera talboti (Allen, 1975)	RFA			2.8	r11
Genus *Dascyllus*					
*Dascyllus aruanus* (Linnaeus, 1758)	RFA			3.3	r1,r8,r11
*Dascyllus marginatus* (Rüppell, 1829)	RFA			2.7	r1
*Dascyllus reticulatus* (Richardson, 1846)	RFA			3.1	r6
*Dascyllus trimaculatus* (Rüppell, 1829)	RFA			2.8	r1,r6,r11
*Dascyllus reticulatus* (Richardson, 1846)	RFA			3.1	r9,r10,r11
Genus *Dischistodus*					
*Dischistodus melanotus* (Bleeker, 1858)	RFA			2.0	r1,r6,r8.r11
*Dischistodus perspicillatus* (Cuvier, 1830)	RFA			2.0	r1,r6,r8,r11
*Dischistodus pseudochrysopoecilus (Allen & Robertson, 1974)	RFA			2.0	r11
*Dischistodus prosopotaenia* (Bleeker, 1852)				2.7	r8,r11
*Dischistodus chrysopoecilus (Schlegel & Müller, 1839)	RFA				r11
Genus *Hemiglyphidodon*					
*Hemiglyphidodon plagiometopon* (Bleeker, 1852)	RFA			2.0	r1
Genus *Neoglyphidodon*					
*Neoglyphidodon melas* (Cuvier, 1830)	RFA			3.4	r1,r6
*Neoglyphidodon thoracotaeniatus* (Fowler & Bean, 1928)	RFA			2.7	r1,r6
Genus *Neopomacentrus*					
*Neopomacentrus azysron* (Bleeker, 1877)	RFA			3.4	r6
Genus Plectroglyphidodon					
*Plectroglyphidodon dickii* (Liénard, 1839)	RFA			3.7	r1,r6,r11
*Plectroglyphidodon lacrymatus* (Quoy & Gaimard, 1825)	RFA			2.2	r1,r6,r8,r11
*Plectroglyphidodon leucozonus* (Bleeker, 1859)	RFA			2.0	r8
Genus *Pomacentrus*					
*Pomacentrus brachialis* Cuvier, 1830	RFA			2.6	r1
*Pomacentrus coelestis* Jordan & Starks, 1901	RFA			3.2	r6,r8,r11
*Pomacentrus moluccensis* Bleeker, 1853	RFA			2.4	r1,r6,r8,r11
*Pomacentrus pavo* (Bloch, 1787)	RFA			3.0	r1,r11
*Pomacentrus philippinus* Evermann & Seale, 1907	RFA			2.7	r1,r6,r8,r11
*Pomacentrus tripunctatus* Cuvier, 1830	RFA			2.0	r1
*Pomacentrus vaiuli* Jordan & Seale, 1906	RFA			3.1	r8,r11
*Pomacentrus bankanensis* Bleeker, 1854	RFA			2.7	r8,r11
Genus *Pomachromis*					
*Pomachromis richardsoni* (Snyder, 1909)	RFA			3.0	r1
Genus *Stegastes*					
*Stegastes albifasciatus* (Schlegel & Müller, 1839)	RFA			2.0	r1,r8
*Stegastes fasciolatus* (Ogilby, 1889)	RFA			2.2	r1,r8,r11
*Stegastes lividus* (Forster, 1801)	RFA				r1
*Stegastes insularis Allen & Emery, 1985	RFA			2.0	r11
*Stegastes lividus* (Forster, 1801)	RFA			2.0	r1
*Stegastes nigricans* (Lacepède, 1802)	RFA	Traumatogenic		2.2	r1
Family Labridae					
Genus *Anampses*					
*Anampses caeruleopunctatus* Rüppell, 1829	RFA			3.4	r1
*Anampses melanurus* Bleeker, 1857	RFA		LC	3.4	r1,r11
*Anampses meleagrides* Valenciennes, 1840	RFA		LC	3.5	r1
*Anampses twistii* Bleeker, 1856	RFA		LC	3.5	r1
Genus *Bodianus*					
*Bodianus axillaris* (Bennett, 1832)	RFA		LC	3.4	r1,r11
*Bodianus bilunulatus* (Lacepède, 1801)	RFA		LC	3.4	r1,r8
*Bodianus loxozonus* (Snyder, 1908)	RFA		LC	3.6	r6
*Bodianus macrourus* (Lacepède, 1801)	RFA		LC	3.5	r1
*Bodianus mesothorax* (Bloch & Schneider, 1801)	RFA		LC	3.2	r6
*Bodianus oxycephalus* (Bleeker, 1862)	RFA			3.5	r1
Genus *Cheilinus*					
*Cheilinus chlorourus* (Bloch, 1791)	RFA		LC	3.5	r1,r6
*Cheilinus fasciatus* (Bloch, 1791)	RFA		LC	3.4	r1,r6,r8
*Cheilinus oxycephalus* Bleeker, 1853	RFA		LC	3.4	r1
*Cheilinus trilobatus* Lacepède, 1801	RFA		LC	3.9	r1,r11
*Cheilinus undulatus* Rüppell, 1835	RFA	Reports of ciguatera poisoning	EN	4.0	r1,r11
Genus *Cheilio*					
*Cheilio inermis* (Forsskål, 1775)	RFA		LC	3.5	r1
*Choerodon melanostigma* Fowler & Bean, 1928	RFA		LC	3.5	r1
*Choerodon schoenleinii* (Valenciennes, 1839)	RFA		NT	3.4	r1
Genus *Cirrhilabrus*					
*Cirrhilabrus melanomarginatus* Randall & Shen, 1978	RFA		LC	3.4	r6,r11
*Cirrhilabrus solorensis* Bleeker, 1853	RFA			3.4	r1
*Cirrhilabrus brunneus* Allen, 2006				3.2	r1
*Cirrhilabrus cyanopleura* (Bleeker, 1851)	RFA			3.4	r9,r11
*Cirrhilabrus exquisitus Smith, 1957	RFA			3.4	r10,r11
Genus *Coris*					
*Coris gaimard* (Quoy & Gaimard, 1824)	RFA	Reports of ciguatera poisoning	LC	3.5	r1,r11
*Coris aygula Lacepède, 1801	RFA		LC	3.7	r11
*Coris dorsomacula* Fowler, 1908	RFA		LC	3.5	r9,r10,r11
Genus *Cymolutes*					
*Cymolutes lecluse* (Quoy & Gaimard, 1824)	RFA		LC	4.2	r1
Genus *Epibulus*					
*Epibulus insidiator* (Pallas, 1770)	RFA	Reports of ciguatera poisoning	LC	4.0	r1,r8,r11
Genus *Gomphosus*					
*Gomphosus varius* Lacepède, 1801	RFA		LC	3.7	r1,r11
Genus *Halichoeres*					
*Halichoeres biocellatus* Schultz, 1960	RFA		LC	3.4	r11
*Halichoeres chrysus* Randall, 1981	RFA		LC	3.4	r6
*Halichoeres hartzfeldii* (Bleeker, 1852)	RFA		LC	3.5	r1
*Halichoeres hortulanus* (Lacepède, 1801)	RFA		LC	3.4	r1,r6,r8,r11
*Halichoeres margaritaceus* (Valenciennes, 1839)	RFA		LC	3.7	r1,r11
*Halichoeres marginatus* Rüppell, 1835	RFA		LC	3.2	r1,r11
*Halichoeres prosopeion* (Bleeker, 1853)	RFA		LC	3.5	r3,r11
*Halichoeres nigrescens* (Bloch & Schneider, 1801)	RFA		LC	3.4	r1,r11
*Halichoeres melasmapomus Randall, 1981	RFA		LC	3.5	r11
*Halichoeres trimaculatus* (Quoy & Gaimard, 1834)	RFA		LC	3.5	r1,r8.r11
*Halichoeres zeylonicus (Bennett, 1833)	RFA		LC	3.5	r11
Genus *Hemigymnus*					
*Hemigymnus fasciatus* (Bloch, 1792)	RFA		LC	3.5	r1,r6,r11
*Hemigymnus melapterus* (Bloch, 1791)	RFA		LC	3.6	r1,r6,r11
Genus *Hologymnosus*					
*Hologymnosus annulatus* (Lacepède, 1801)	RFA		LC	4.2	r1,r11
*Hologymnosus doliatus* (Lacepède, 1801)	RFA		LC	3.8	r9,r10,r11
Genus *Iniistius*					
*Iniistius aneitensis* (Günther, 1862)	RFA		LC	3.5	r1
*Iniistius melanopus* (Bleeker, 1857)	RFA		LC	3.5	r1
*Iniistius pavo* (Valenciennes, 1840)	RFA		LC	3.5	r1
Genus *Labrichthys*					
*Labrichthys unilineatus* (Guichenot, 1847)	RFA		LC	3.3	r1,r6
Genus *Labroides*					
*Labroides bicolor* Fowler & Bean, 1928	RFA		LC	4.0	r6,r11
*Labroides dimidiatus* (Valenciennes, 1839)	RFA		LC	3.5	r1,r6,r11
Genus *Labropsis*					
*Labropsis manabei* Schmidt, 1931	RFA		LC	3.3	r1,r6
Genus *Macropharyngodon*					
*Macropharyngodon meleagris* (Valenciennes, 1839)	RFA		LC	3.1	r1,r8,r11
Genus *Novaculichthys*					
*Novaculichthys taeniourus* (Lacepède, 1801)	RFA		LC	3.3	r1
Genus *Oxycheilinus*					
*Oxycheilinus celebicus* (Bleeker, 1853)	RFA		LC	3.8	r1,r11
*Oxycheilinus digramma* (Lacepède, 1801)	RFA		LC	3.7	r1,r11
*Oxycheilinus mentalis* (Rüppell, 1828)	RFA		LC	3.8	r1
*Oxycheilinus orientalis* (Günther, 1862)	RFA		LC	3.8	r1
*Oxycheilinus unifasciatus* (Streets, 1877)	RFA	Reports of ciguatera poisoning	LC	4.1	r6,r11
*Oxycheilinus bimaculatus* (Valenciennes, 1840)	RFA		LC	3.5	r9,r11
Genus *Pseudocheilinus*					
*Pseudocheilinus evanidus* Jordan & Evermann, 1903	RFA		LC	3.5	r11
*Pseudocheilinus hexataenia* (Bleeker, 1857)	RFA		LC	3.2	r1,r6
Genus *Pseudocoris*					
*Pseudocoris yamashiroi* (Schmidt, 1931)	RFA		LC	3.4	r1
Genus *Pteragogus*					
*Pteragogus flagellifer* (Valenciennes, 1839)	RFA		LC	3.5	r1
Genus *Stethojulis*					
*Stethojulis balteata* (Quoy & Gaimard, 1824)	RFA		LC	3.5	r1
*Stethojulis bandanensis* (Bleeker, 1851)	RFA		LC	3.2	r1,r11
*Stethojulis interrupta* (Bleeker, 1851)	RFA		LC	3.4	r1,r8
*Stethojulis strigiventer* (Bennett, 1833)	RFA		LC	3.1	r1,r11
Genus *Thalassoma*					
*Thalassoma cupido* (Temminck & Schlegel, 1845)	DEM		LC	3.5	r6
*Thalassoma hardwicke* (Bennett, 1830)	RFA		LC	3.5	r1,r6,r8,r11
*Thalassoma lunare* (Linnaeus, 1758)	RFA		LC	3.5	r1,r6,r11
*Thalassoma purpureum* (Forsskål, 1775)	RFA		LC	3.8	r1,r6
*Thalassoma quinquevittatum* (Lay & Bennett, 1839)	RFA		LC	3.6	r1,r6,r11
*Thalassoma trilobatum* (Lacepède, 1801)	RFA		LC	3.8	r1,r6
*Thalassoma lutescens* (Lay & Bennett, 1839)	RFA		LC	3.7	r9,r10,r11
*Thalassoma amblycephalum* (Bleeker, 1856)	RFA		LC	3.1	r9,r10,r11
Family Scaridae					
Genus *Calotomus*					
*Calotomus japonicus* (Valenciennes, 1840)	RFA		LC	2.0	r1
*Calotomus spinidens* (Quoy & Gaimard, 1824)	RFA		LC	2.0	r1
Genus *Cetoscarus*					
*Cetoscarus bicolor* (Rüppell, 1829)	RFA		LC	2.0	r1,r11
Genus *Chlorurus*					
*Chlorurus microrhinos* (Bleeker, 1854)	RFA	Reports of ciguatera poisoning	LC	2.6	r1
*Chlorurus sordidus* (Forsskål, 1775)	RFA; OD	Reports of ciguatera poisoning	LC	2.6	r1,r6,r7,r8,r11
*Chlorurus gibbus* (Rüppell, 1829)	RFA	Reports of ciguatera poisoning	LC	2.0	r1
*Chlorurus bleekeri* (de Beaufort, 1940)	RFA		LC	2.0	r10,r11
*Chlorurus japanensis* (Bloch, 1789)	RFA		LC	2.0	r6,r9,r10,r11
Genus *Hipposcarus*					
*Hipposcarus longiceps* (Valenciennes, 1840)	RFA		LC	2.0	r1
Genus *Leptoscarus*					
*Leptoscarus vaigiensis* (Quoy & Gaimard, 1824)	RFA		LC	2.0	r1
Genus *Scarus*					
*Scarus dimidiatus* Bleeker, 1859	RFA		LC	2.0	r1,r8,r11
*Scarus ferrugineus* Forsskål, 1775	RFA		LC	2.0	r1,r7
*Scarus festivus* Valenciennes, 1840	RFA		LC	2.0	r1,r11
*Scarus forsteni* (Bleeker, 1861)	RFA		LC	2.0	r1,r8,r11
*Scarus frenatus* Lacepède, 1802	RFA		LC	2.0	r1,r11
*Scarus ghobban* Forsskål, 1775	RFA		LC	2.0	r1
*Scarus globiceps* Valenciennes, 1840	RFA		LC	2.0	r1,r11
*Scarus niger* Forsskål, 1775	RFA		LC	2.0	r1,r11
*Scarus oviceps* Valenciennes, 1840	RFA		LC	2.0	r1,r7,r11
*Scarus prasiognathos* Valenciennes, 1840	RFA		LC	2.0	r1,r7
*Scarus psittacus* Forsskål, 1775	RFA		LC	2.0	r1,r7,r11
*Scarus rivulatus* Valenciennes, 1840	RFA		LC	2.0	r1,r11
*Scarus rubroviolaceus* Bleeker, 1847	RFA		LC	2.0	r1
*Scarus scaber* Valenciennes, 1840	RFA		LC	2.0	r1
*Scarus tricolor* Bleeker, 1847	RFA		LC	2.0	r1,r7
*Scarus schlegeli* (Bleeker, 1861)	RFA		LC	2.0	r9.r10,r11
Family Pinguipedidae					
Genus *Parapercis*					
*Parapercis clathrata* Ogilby, 1910	RFA			3.6	r1,r11
*Parapercis cylindrica* (Bloch, 1792)	RFA			3.0	r1,r8,r11
*Parapercis pacifica* Imamura & Yoshino, 2007	RFA			3.6	r1,r6,r8,r11
*Parapercis millepunctata (Günther, 1860)	RFA			3.5	r11
*Parapercis xanthozona (Bleeker, 1849)	RFA		LC	3.6	r11
*Parapercis hexophtalma* (Cuvier, 1829)	RFA			3.6	r11
Family Uranoscopidae					
Genus *Uranoscopidae*					
*Uranoscopus japonicus* Houttuyn, 1782	BAD	Venomous	LC	4.0	r1
Family Tripterygiidae					
Genus *Helcogramma*					
*Helcogramma chica Rosenblatt, 1960	RFA		LC	3.0	r11
Family Blenniidae					
Genus *Aspidontus*					
*Aspidontus tractus* Fowler, 1903	RFA			2.9	r1
*Aspidontus taeniatus* Quoy & Gaimard, 1834				3.8	r8,r11
Genus *Blenniella*					
*Blenniella periophthalmus* (Valenciennes, 1836)	RFA		LC	3.3	r1
Genus *Cirripectes*					
*Cirripectes castaneus* (Valenciennes, 1836)	RFA		LC	2.0	r6,r11
*Cirripectes variolosus* (Valenciennes, 1836)	RFA		LC	2.0	r1
Genus *Entomacrodus*					
*Entomacrodus caudofasciatus* (Regan, 1909)	RFA		LC	2.0	r1
Genus *Istiblennius*					
*Istiblennius dussumieri* (Valenciennes, 1836)	RFA		LC	2.0	r1
*Istiblennius edentulus* (Forster & Schneider, 1801)	RFA		LC	2.0	r1
*Istiblennius lineatus* (Valenciennes, 1836)	RFA		LC	2.0	r1
Genus *Meiacanthus*					
*Meiacanthus atrodorsalis* (Günther, 1877)	RFA	Venomous	LC	3.5	r6
Genus *Plagiotremus*					
*Plagiotremus rhinorhynchos* (Bleeker, 1852)	RFA		LC	4.5	r6,r11
*Plagiotremus tapeinosoma* (Bleeker, 1857)	RFA	Other	LC	3.8	r6,r11
Genus *Salarias*					
*Salarias fasciatus* (Bloch, 1786)	RFA		LC	2.0	r1
Genus *praealticus*					
*Praealticus margaritatus* (Kendall & Radcliffe, 1912)	PN		LC	2.0	r1
Family Ptereleotridae					
Genus *Nemateleotris*					
*Nemateleotris magnifica* Fowler, 1938	RFA		LC	3.1	r6,r11
Genus *Ptereleotris*					
*Ptereleotris evides* (Jordan & Hubbs, 1925)	RFA		LC	3.4	r6,r11
Family Gobiidae					
Genus *Acentrogobius*					
*Acentrogobius caninus* (Valenciennes, 1837)	DEM; AMP	Poisonous to eat	LC	3.5	r1
Genus *Amblygobius*					
*Amblygobius albimaculatus* (Rüppell, 1830)	RFA			2.6	r1
*Amblygobius nocturnus (Herre, 1945)	RFA			2.7	r11
*Amblygobius phalaena* (Valenciennes, 1837)	RFA			3.6	r9,r11
Genus *Asterropteryx*					
*Asterropteryx semipunctata* Rüppell, 1830	RFA			2.4	r1
Genus *Bathygobius*					
*Bathygobius fuscus* (Rüppell, 1830)	RFA		LC	3.4	r1
Genus *Callogobius*					
*Callogobius sclateri* (Steindachner, 1879)	RFA			3.3	r1
Genus *Ctenogobiops*					
*Ctenogobiops feroculus* Lubbock & Polunin, 1977	RFA		LC	3.4	r8
Genus *Eviota*					
*Eviota abax* (Jordan & Snyder, 1901)	RFA			3.2	r1
*Eviota prasites* Jordan & Seale, 1906	RFA		LC	3.1	r9,r11
Genus *Gobiodon*					
*Gobiodon erythrospilus* Bleeker, 1875	RFA			3.4	r1
*Gobiodon multilineatus* Wu, 1979	RFA			3.2	r1
*Gobiodon oculolineatus* Wu, 1979	RFA			3.2	r1
*Gobiodon okinawae* Sawada, Arai & Abe, 1972	RFA			3.2	r1
*Gobiodon quinquestrigatus* (Valenciennes, 1837)	RFA			3.4	r1
Genus *Istigobius*					
*Istigobius ornatus* (Rüppell, 1830)	RFA		LC	3.5	r1
Genus *Paragobiodon*					
*Paragobiodon echinocephalus* (Rüppell, 1830)	RFA		LC	3.2	r1
*Paragobiodon melanosoma* (Bleeker, 1853)	RFA		LC	3.1	r1
*Paragobiodon xanthosoma* (Bleeker, 1853)	RFA		LC	3.2	r1
Genus *Priolepis*					
*Priolepis semidoliata* (Valenciennes, 1837)	RFA		LC	3.1	r1
Genus *Valenciennea*					
*Valenciennea longipinnis* (Lay & Bennett, 1839)	RFA			3.0	r1
*Valenciennea strigata* (Broussonet, 1782)	RFA			4.0	r1,r6,r11
Family Ephippidae					
Genus *Platax*					
*Platax orbicularis* (Forsskål, 1775)	RFA		LC	3.3	r2
*Platax teira* (Forsskål, 1775)	RFA; AMP		LC	4.0	r2
Family Siganidae					
Genus *Siganus*					
*Siganus argenteus* (Quoy & Gaimard, 1825)	RFA	Venomous	LC	2.0	r1
*Siganus canaliculatus* (Park, 1797)	RFA; OD	Venomous	LC	2.8	r1
*Siganus corallinus* (Valenciennes, 1835)	RFA	Venomous	LC	2.0	r1
*Siganus fuscescens* (Houttuyn, 1782)	RFA	Venomous	LC	2.0	r1
*Siganus guttatus* (Bloch, 1787)	RFA	Venomous	LC	2.7	r1,r7
*Siganus puellus* (Schlegel, 1852)	RFA	Venomous	LC	3.0	r1,r11
*Siganus punctatus* (Schneider & Forster, 1801)	RFA	Venomous	LC	2.0	r1,r11
*Siganus spinus* (Linnaeus, 1758)	RFA	Venomous	LC	2.0	r1
*Siganus virgatus* (Valenciennes, 1835)	RFA	Venomous	LC	2.7	r2
*Siganus vulpinus* (Schlegel & Müller, 1845)	RFA	Venomous	LC	2.7	r1,r6
Family Acanthuridae					
Genus *Ctenochaetus*					
*Ctenochaetus striatus* (Quoy & Gaimard, 1825)	RFA	Reports of ciguatera poisoning	LC	2.0	r1,r6,r8,r11
*Ctenochaetus cyanocheilus* Randall & Clements, 2001	RFA		LC	2.0	r9,r11
Genuus *Naso*					
*Naso annulatus* (Quoy & Gaimard, 1825)	RFA	Reports of ciguatera poisoning	LC	2.1	r3
*Naso brachycentron* (Valenciennes, 1835)	RFA		LC	2.7	r2
*Naso brevirostris* (Cuvier, 1829)	RFA	Reports of ciguatera poisoning	LC	2.2	r1,r11
*Naso hexacanthus* (Bleeker, 1855)	RFA		LC	3.1	r1,r7,r11
*Naso lituratus* (Forster, 1801)	RFA	Venomous	LC	2.3	r1,r6
*Naso lopezi* Herre, 1927	RFA		LC	2.9	r2
*Naso thynnoides* (Cuvier, 1829)	RFA		LC	3.0	r1,r6
*Naso unicornis* (Forsskål, 1775)	RFA	Reports of ciguatera poisoning	LC	2.2	r1
*Naso vlamingii* (Valenciennes, 1835)	RFA		LC	2.2	r1
Genus *Prionurus*					
*Prionurus scalprum* Valenciennes, 1835	RFA	Venomous		2.0	r1,r7
Genus *Zebrasoma*					
*Zebrasoma flavescens* (Bennett, 1828)	RFA		LC	2.0	r1,r8
*Zebrasoma scopas* (Cuvier, 1829)	RFA		LC	2.0	r6,r8,r11
*Zebrasoma velifer* (Bloch, 1795)	RFA		LC	2.0	r1,r6,r8,r11
Family Zanclidae					
Genus *Zanclus*					
*Zanclus cornutus* (Linnaeus, 1758)	RFA		LC	2.5	r1,r6,r8,r11
Family Acanthuridae					
Genus *Acanthurus*					
*Acanthurus dussumieri* Valenciennes, 1835	RFA		LC	2.0	r1
*Acanthurus gahhm* (Forsskål, 1775)	RFA		LC	2.0	r4
*Acanthurus japonicus* (Schmidt, 1931)	RFA		LC	2.0	r6,r11
*Acanthurus lineatus* (Linnaeus, 1758)	RFA	Venomous	LC	2.0	r1
*Acanthurus mata* (Cuvier, 1829)	RFA	Venomous	LC	2.5	r1
*Acanthurus nigricans* (Linnaeus, 1758)	RFA		LC	2.0	r2
*Acanthurus nigrofuscus* (Forsskål, 1775)	RFA	Reports of ciguatera poisoning	LC	2.0	r1,r7,r11
*Acanthurus olivaceus* Bloch & Schneider, 1801	RFA		LC	2.3	r1,r6,r11
*Acanthurus thompsoni* (Fowler, 1923)	RFA		LC	3.6	r1
*Acanthurus triostegus* (Linnaeus, 1758)	RFA	Reports of ciguatera poisoning	LC	2.8	r1,r6,r11
*Acanthurus xanthopterus* Valenciennes, 1835	RFA	Venomous	LC	2.9	r2
Family Sphyraenidae					
Genus *Sphyraena*					
*Sphyraena barracuda* (Edwards, 1771)	RFA	Traumatogenic	LC	4.5	r1,r7
*Sphyraena forsteri* Cuvier, 1829	RFA	Reports of ciguatera poisoning		4.4	r1,r7
*Sphyraena helleri* Jenkins, 1901	RFA			4.5	r1
*Sphyraena obtusata* Cuvier, 1829	RFA			4.5	r1
Family Gempylidae					
Genus *Gempylus*					
*Gempylus serpens* Cuvier, 1829	PEL; OD		LC	4.4	r1
Genus *Lepidocybium*					
*Lepidocybium flavobrunneum* (Smith, 1843)	BEP; OD		LC	4.3	r1
Genus *Promethichthys*					
*Promethichthys prometheus* (Cuvier, 1832)	BEP; OD	Reports of ciguatera poisoning	LC	4.2	r1
Genus *Rexea*					
*Rexea prometheoides* (Bleeker, 1856)	BEP			4.2	r3
Genus *Ruvettus*					
*Ruvettus pretiosus* Cocco, 1833	BEP; OD	Poisonous to eat	LC	4.2	r1,r7
Genus *Thyrsitoides*					
*Thyrsitoides marleyi* Fowler, 1929	BEP			4.2	r1,r7
Family Scombridae					
Genus *Acanthocybium*					
*Acanthocybium solandri* (Cuvier, 1832)	PEL; OD	Reports of ciguatera poisoning	LC	4.3	r1
Genus *Auxis*					
*Auxis thazard* (Lacepède, 1800)	PN; OD		LC	4.4	r1
Genus *Euthynnus*					
*Euthynnus affinis* (Cantor, 1849)	PN; OD	Reports of ciguatera poisoning	LC	4.5	r1
Genus *Grammatorcynus*					
*Grammatorcynus bicarinatus* (Quoy & Gaimard, 1825)	RFA; OD		LC	4.5	r1
Genus *Gymnosarda*					
*Gymnosarda unicolor* (Rüppell, 1836)	RFA; OD	Reports of ciguatera poisoning	LC	4.5	r1
Genus *Katsuwonus*					
*Katsuwonus pelamis* (Linnaeus, 1758)	PEL; OD	Reports of ciguatera poisoning	LC	4.4	r1
Genus *Rastrelliger*					
*Rastrelliger kanagurta* (Cuvier, 1816)	PN; OD			3.2	r1
Genus *Thunnus*					
*Thunnus albacares* (Bonnaterre, 1788)	PEL; OD		NT	4.4	r1
*Thunnus obesus* (Lowe, 1839)	PEL; OD		VU	4.5	r1
Family Xiphiidae					
Genus *Xiphias*					
*Xiphias gladius* Linnaeus, 1758	PEL; OD		EN	4.5	r1
Family Istiophoridae					
Genus *Histiophofus*					
*Istiophorus platypterus* (Shaw, 1792)	PEL; OD		LC	4.5	r1
Genus *Istiompax*					
*Istiompax indica* (Cuvier, 1832)	PEL; OD			4.5	r1
Family Psettodidae					
Genus *Psettodes*					
*Psettodes erumei* (Bloch & Schneider, 1801)	DEM			4.4	r1
Family Bothidae					
Genus *Bothus*					
*Bothus mancus* (Broussonet, 1782)	RFA		LC	4.4	r1
*Bothus pantherinus* (Rüppell, 1830)	RFA		LC	3.5	r1
Family Soleidae					
Genus *Aseraggodes*					
*Aseraggodes dubius* Weber, 1913	DEM			3.5	r5
Order Tetraodontiformes					
Family Balistidae					
Genus *Abalistes*					
*Abalistes stellatus* (Anonymous, 1798)	RFA			3.4	r1,r7
Genus *Balistapus*					
*Balistapus undulatus* (Park, 1797)				3.4	r1
Genus *Balistes*					
*Balistes rotundatus* Marion de Procé, 1822	RFA	Traumatogenic		3.5	r1
Genus *Balistoides*					
*Balistoides conspicillum* (Bloch & Schneider, 1801)	RFA	Reports of ciguatera poisoning		3.3	r1
*Balistoides viridescens* (Bloch & Schneider, 1801)	RFA	Reports of ciguatera poisoning		3.3	r2
Genus *Melichthys*					
*Melichthys niger* (Bloch, 1786)	RFA		LC	2.4	r2
*Melichthys vidua* (Richardson, 1845)	RFA			3.4	r1,r11
Genus *Odonus*					
*Odonus niger* (Rüppell, 1836)	RFA			3.2	r1
Genus *Pseudobalistes*					
*Pseudobalistes flavimarginatus* (Rüppell, 1829)	RFA	Reports of ciguatera poisoning		2.8	r1
*Pseudobalistes fuscus* (Bloch & Schneider, 1801)	RFA			4.0	r1
Genus *Rhinecanthus*					
*Rhinecanthus aculeatus* (Linnaeus, 1758)	RFA			3.2	r1,r11
*Rhinecanthus rectangulus* (Bloch & Schneider, 1801)	RFA			3.5	r1,r11
Genus *Sufflamen*					
*Sufflamen chrysopterum* (Bloch & Schneider, 1801)	RFA			3.5	r1,r11
*Sufflamen fraenatum* (Latreille, 1804)	RFA; OD		LC	3.7	r3,r6
Genus *Xanthichthys*					
*Xanthichthys lineopunctatus* (Hollard, 1854)	RFA			3.5	r1
*Xanthichthys auromarginatus* (Bennett, 1832)	RFA			3.0	r9,r11
Family Monacanthidae					
Genus *Aluterus*					
*Aluterus scriptus* (Osbeck, 1765)	RFA	Reports of ciguatera poisoning	LC	2.8	r1
Genus *Cantherhines*					
*Cantherhines dumerilii* (Hollard, 1854)	RFA		LC	3.1	r1,r6,r11
*Cantherhines pardalis* (Rüppell, 1837)	RFA		LC	3.5	r1,r11
Genus *Chaetodermis*					
*Chaetodermis penicilligerus* (Cuvier, 1816)	RFA		LC	2.8	r1
Genus *Oxymonacanthus*					
*Oxymonacanthus longirostris* (Bloch & Schneider, 1801)	RFA		VU	3.3	r1,r6
Genus *Paraluteres*					
*Paraluteres prionurus* (Bleeker, 1851)	RFA		LC	2.7	r6
Genus *Pervagor*					
*Pervagor janthinosoma* (Bleeker, 1854)	RFA		LC	2.9	r1
*Pervagor melanocephalus* (Bleeker, 1853)	RFA		LC	2.9	r1,r6
Family Ostraciidae					
Genus *Lactoria*					
*Lactoria cornuta* (Linnaeus, 1758)	RFA	Reports of ciguatera poisoning		3.5	r1
Genus *Ostracion*					
*Ostracion cubicus* Linnaeus, 1758	RFA			3.4	r1,r6
*Ostracion meleagris* Shaw, 1796	RFA	Venomous		2.7	r2
*Ostracion rhinorhynchos* Bleeker, 1851	RFA	Venomous		3.5	r2
Family Tetraodontidae					
Genus *Arothron*					
*Arothron hispidus* (Linnaeus, 1758)	RFA	Poisonous to eat	LC	3.2	r1
*Arothron meleagris* (Anonymous, 1798)	RFA	Poisonous to eat	LC	3.6	r1
*Arothron nigropunctatus* (Bloch & Schneider, 1801)	RFA	Poisonous to eat	LC	3.4	r1,r6,r11
*Arothron stellatus* (Anonymous, 1798)	RFA	Poisonous to eat	LC	3.7	r2
Genus *Canthigaster*					
*Canthigaster jactator* (Jenkins, 1901)	RFA		LC	2.8	r1
*Canthigaster rivulata* (Temminck & Schlegel, 1850)	RFA	Venomous	LC	3.1	r1
*Canthigaster valentini* (Bleeker, 1853)	RFA	Poisonous to eat	LC	2.8	r6,r11
*Canthigaster coronata* (Vaillant & Sauvage, 1875)	Rfa	Other	LC	3.5	r10,r11
Genus *Lagocephalus*					
*Lagocephalus lagocephalus* (Linnaeus, 1758)	BEP; OD	Other	LC	3.7	r1
*Lagocephalus sceleratus* (Gmelin, 1789)	RFA	Poisonous to eat	LC	3.7	r1
Genus *Sphoeroides*					
*Sphoeroides pachygaster* (Müller & Troschel, 1848)	BEP		VU	4.2	r3
Family Diodontidae					
Genus *Chilomycterus*					
*Chilomycterus reticulatus* (Linnaeus, 1758)	RFA	Venomous	LC	3.5	r2
Genus *Diodon*					
*Diodon holocanthus* Linnaeus, 1758	RFA	Reports of ciguatera poisoning	LC	3.9	r1
*Diodon hystrix* Linnaeus, 1758	RFA	Poisonous to eat	LC	3.7	r1
*Diodon liturosus* Shaw, 1804	RFA	Reports of ciguatera poisoning		3.5	r2
